# Effect Analysis of Design Variables on the Disc in a Double-Eccentric Butterfly Valve

**DOI:** 10.1155/2014/305085

**Published:** 2014-04-17

**Authors:** Sangmo Kang, Da-Eun Kim, Kuk-Kyeom Kim, Jun-Oh Kim

**Affiliations:** Department of Mechanical Engineering, Dong-A University, Busan 604-714, Republic of Korea

## Abstract

We have performed a shape optimization of the disc in an industrial double-eccentric butterfly valve using the effect analysis of design variables to enhance the valve performance. For the optimization, we select three performance quantities such as pressure drop, maximum stress, and mass (weight) as the responses and three dimensions regarding the disc shape as the design variables. Subsequently, we compose a layout of orthogonal array (L16) by performing numerical simulations on the flow and structure using a commercial package, ANSYS v13.0, and then make an effect analysis of the design variables on the responses using the design of experiments. Finally, we formulate a multiobjective function consisting of the three responses and then propose an optimal combination of the design variables to maximize the valve performance. Simulation results show that the disc thickness makes the most significant effect on the performance and the optimal design provides better performance than the initial design.

## 1. Introduction

The design process of a valve system demands a variety of requirements such as low pressure drop, no leakage, high structural stability, and low manufacturing cost. Such design requirements indicate conditions or performances needed by the whole or a part of the system to meet certain design criteria. A butterfly valve is generally used for isolating or regulating flow and, particularly, allows for quick shutoff of flow. In the present study, we want to discuss a double-eccentric butterfly valve that is being widely used at the industrial sites due to the small space taken up by it and its small weight and low maintenance cost. Note that the standard pressure grades (ISO 10631, ASME B16.34, AWWA C504, KS B 2333, and so on), inspection methods, and pressure tests (ISO 5208, KS B 2304, and so on) are currently used in accordance with the design requirements for the use of butterfly valves in the industrial field.

Ding et al. [1] optimized a sealing structure in a triple-eccentric butterfly valve, while Ejab and Samir [2] optimized a butterfly valve housing to reduce its weight. Since then, a variety of optimal design techniques have been applied to enhance the performance and stability of industrial valves [3–6]. In this context, the present study aims to select design variables needed to optimize an industrial double-eccentric butterfly valve, analyze the effect of the design variables on the valve performance, and then propose an optimal combination of the design variables to provide the best performance.

## 2. Flow and Structural Analyses

### 2.1. Analysis Model: Butterfly Valve

**Figure 1:**
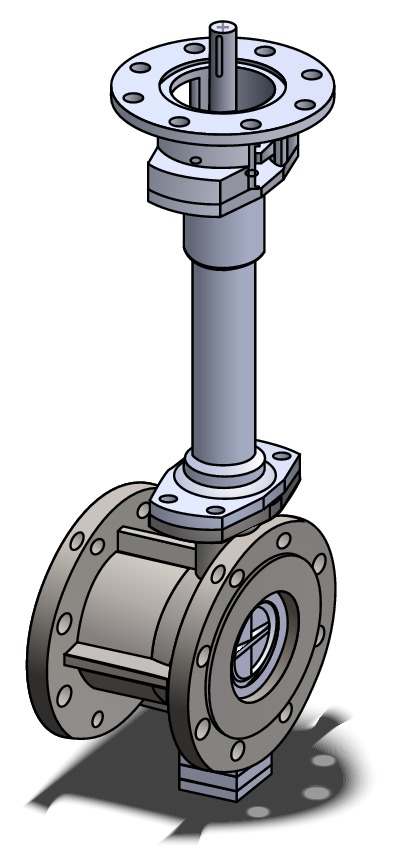
External appearance of the double-eccentric butterfly valve considered in the present study.

Figure 1 shows the external appearance of the industrial butterfly valve considered in the present study, which is drawn using a commercial 3D modeling package, Solidworks. The valve consists mainly of disc, shaft, body, and bush. The opening and closing operation of the valve is run by rotating the disc, which is positioned in the center of the pipe. The valve is called the double-eccentric butterfly valve because of the following two offsets on the valve; the shaft of the disc is offset from the center line of the disc seat and body seal (offset one) and from the center line of the bore (offset two). Therefore, it has much less wear than the concentric butterfly valve and is excellent in the flow control [3]. In the present study, we attempt to perform a shape optimization of the disc installed in a double-eccentric butterfly valve using the effect analysis of design variables to enhance the valve performance.

For the optimization of the disc, we select three performance quantities such as pressure drop, maximum stress, and mass (weight) as the responses and three dimensions regarding the disc shape as the design variables. Subsequently, we compose a layout of orthogonal array (L16) by performing numerical simulations on the flow and structure of the valve using a commercial package, ANSYS v13.0.

### 2.2. Flow Analysis

**Figure 2:**
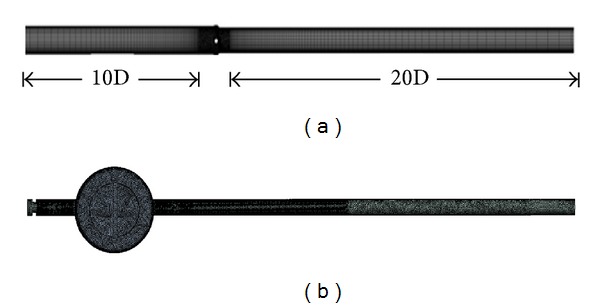
Models and grid systems generated for the structural and flow analyses: (a) flow and (b) structure.

Figure 2(a) shows the computational domain constructed for the flow analysis and the grid system created using a commercial package, ANSYS v13.0. The domain is composed of the valve and the inlet and outlet pipes connected to it on both sides. The pipes have lengths of 10*D* and 20*D* (*D* is the pipe diameter), respectively, so that the flow becomes fully developed or stabilized. To calculate the pressure drop across the valve, the pressures are measured at 2*D* and 6*D* positions, respectively, before and behind the valve. For the turbulence model, the shear stress transport (SST) model is used. The SST model is a combination of *k*-*ω* and *k*-*ε* models produced to make the best use of the merit of each of both models; the former is more accurate near the wall, whereas the latter is away from the wall [7]. The working conditions applied to the flow analysis are shown in Table 1.

**Figure 3:**
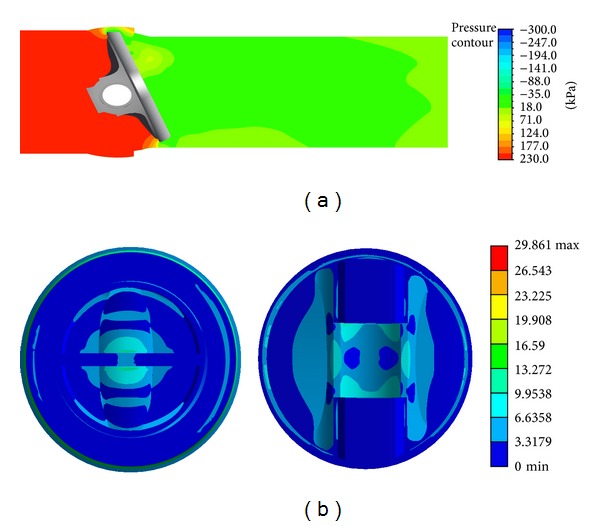
Results of the numerical simulations on the flow and structure of the initial model: (a) pressure distribution around the valve and (b) stress distribution on the valve disc.

At first, we perform a numerical simulation on the flow through the initial design (model) of the valve and then present one of its results in Figure 3(a). Figure 3(a) shows the pressure distribution generated around the valve for the initial model. It is observed that the pressure sharply drops across the valve and then gradually recovers downstream behind it.

### 2.3. Structural Analysis

Figure 2(b) shows the model and grid system used for the structural analysis. The maximum allowable working pressure or design pressure, 1 MPa, is applied to the back of the valve disc as a boundary condition (see Table 1) and the constraint conditions are given to the top and bottom of the valve shaft and the contact surface (or the metal seat on the valve disc) between the disc and body. To reduce the analysis time, we do not attempt to perform the structural analysis on the whole valve but instead on each of the components comprising the valve. In other words, we establish the boundary conditions and constraints by following the studies of Shin [8] executed on the butterfly valve. The materials necessary for the structural analysis are also presented in Table 1.

Subsequently, we perform a numerical simulation on the structure of the initial model and then present the stress distribution on the valve disc, as shown in Figure 3(b). The maximum stress occurs on the contact surface between the disc and body to which a constraint condition is given.

## 3. Shape Optimization of the Disc

### 3.1. Design of Experiments

**Figure 4:**
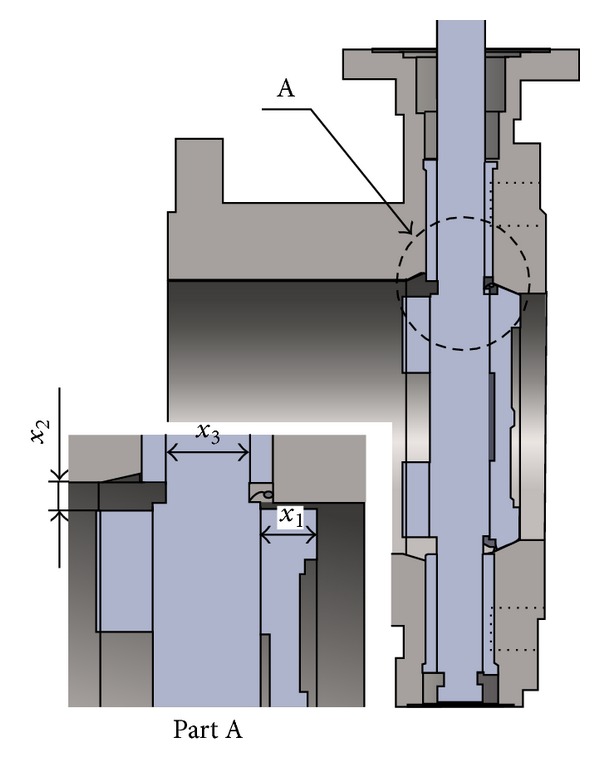
Design variables selected for the shape optimization of the valve disc.

As mentioned above, we chose three performance quantities such as pressure drop, maximum stress, and mass (weight) as the responses for the optimization of the butterfly valve. Next we also select three dimensions regarding the disc shape as the design variables, which are expected to significantly influence the valve performance or the responses.  Figure 4 shows the schematic details on the three design variables, which include the thickness of the valve disc (*x*_1_), the distance between the bush and valve disc boss (*x*_2_), and the diameter of the valve shaft (*x*_3_). To make the design of experiments, four uniformly spaced levels are assigned to each design variable and presented in Table 2. Here, the bottom and top limits (levels 1 and 4) are determined, respectively, through the decrease and increase by 10% from the initial value and then levels 2 and 3 are appropriately distributed with a uniform space.

Subsequently we make the design of experiments based on the L16 orthogonal array to perform the effect analysis of the design variables on the valve performance and then propose their optimal combination. For the effect analysis, we define the multiobjective function consisting of the three responses, pressure drop, maximum stress, and mass, as follows:

(1) $$\begin{array}{c} \Phi =-10\log\left(\frac{1}{n}\overset{n}{\underset{i=1}{\sum }}y_{i}^{2}\right), \end{array}$$

where *y*_*i*_ denotes the response and *n* is the response number (here *n* = 3). In other words, we attempt to determine an optimal combination of the design variables in a manner to maximize the above defined objective function (1).  Table 3 shows the layout of the L16 orthogonal arrays composed for the design of experiments and the responses obtained by the full numerical simulations (flow and structural analyses).

### 3.2. Effect Analysis and Shape Optimization

**Figure 5:**
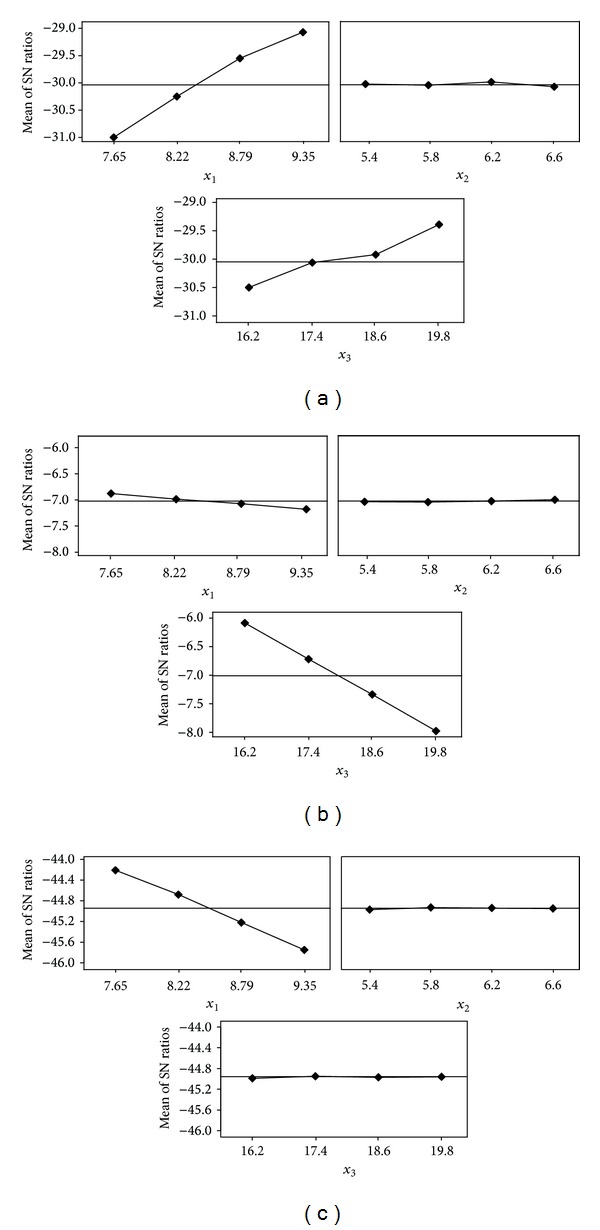
Plots to represent the main effects of the design variables on (a) pressure drop, (b) maximum stress, and (c) mass.

Finally we make the effect analysis of the design variables on the responses and then attempt to propose their optimal combination to enhance the valve performance. Here, the effect analysis is made by evaluating how much each design variable affects the responses according to the level through the analysis of the multiobjective function. The analysis results according to the level are plotted in Figure 5 and tabulated in Tables 4, 5, and 6. Note that such level based effect analysis provides very effective information to determine the design direction [9]. Following the effect analysis results (see Figure 5 and Tables 4–6), we can find that the performance (or responses) can become improved or worsened depending on the level of each design variable. Therefore, we can produce an optimal combination of the design variables by quantifying the effect by the level of each design variable on the performance. In this paper, we calculate the level based effects of the design variables and, as a result, find that the disc thickness (*x*_1_) has the greatest effect on the pressure drop at 95.7% and on the maximum stress at 61.7%, while the shaft diameter (*x*_3_) has an effect on the mass at 84.4%. On the contrary, the distance between the bush and boss (*x*_2_) has a relatively trivial effect on all the responses.

From the effect analysis results, we can draw an optimal combination of the design variables, that is, the disc thickness at 7.65 mm, the distance between the boss and bush at 6.2 mm, and the shaft diameter at 17.4 mm, which is corresponding to the 16th experiment in the orthogonal array table (Table 3).  Table 7 shows a comparison between the initial model and the optimal model; the optimal model is improved by 2.74% in the mass and by 6.16% in the pressure drop compared with the initial model. However, there is no improvement found in the maximum stress, but it is still within the material yield strength, 205 MPa.

## 4. Conclusions

In the present study, we have performed a shape optimization of the disc in an industrial double-eccentric butterfly valve using the effect analysis of design variables to enhance the valve performance. For the optimization, we select three performance quantities such as pressure drop, maximum stress, and mass (weight) as the responses and three dimensions regarding the disc shape as the design variables. Here, the effect analysis is made by evaluating how much each design variable affects the responses according to the level through the design of experiments made with the L16 orthogonal array and the analysis of the multiobjective function.

Results indicate that the disc thickness has the greatest effect on the flow and structural performance of the butterfly valve considered in the present study. Through the shape optimization of the disc using the effect analysis, the performance of the optimal design becomes improved by 6.16% in the pressure drop and by 2.74% in the mass compared with the initial design. On the other hand, there is no improvement found in the maximum stress. Although only one design variable plays a dominant role in the valve performance in this study, systematic design with more diverse variables regarding the disc shape in addition to the disc thickness is expected to bring better findings in later studies.

## Figures and Tables

**Table 1:** Operating conditions for the flow analysis and materials for the structural analysis.

Operating conditions	
Working fluid	water
Working Temperature	20°C
Design pressure	1 MPa

Materials	

Body, disc	CF8M
Shaft, bush	SUS316

**Table 2:** Design variables and their levels.

Design variable	Unit	Level 1	Level 2	Level 3	Level 4
Disc thickness (*x*_1_)	mm	7.65	8.22	8.79	9.35
Distance (*x*_2_)	mm	5.4	5.8	6.2	6.6
Diameter (*x*_3_)	mm	16.2	17.4	18.6	19.8

**Table 3:** Layout of the L16 orthogonal array based on the design of experiments and the responses obtained by the full numerical simulations.

Exp	*x*_1_	*x*_2_	*x*_3_	Stress (MPa)	Mass (kg)	Δ*p* (kPa)
1	7.65	5.4	16.2	36.47	1.98	163.8
2	8.22	5.8	17.4	32.56	2.16	171.0
3	8.79	6.2	18.6	29.22	2.34	182.4
4	9.35	6.6	19.8	26.59	2.54	194.0
5	8.22	6.2	19.8	29.91	2.49	171.6
6	7.65	6.6	18.6	35.82	2.28	162.8
7	9.35	5.4	17.4	28.79	2.21	194.1
8	8.79	5.8	16.2	32.52	2.03	182.7
9	8.79	6.6	17.4	30.63	2.17	182.2
10	9.35	6.2	16.2	30.84	2.05	193.9
11	7.65	5.8	19.8	33.93	2.47	161.8
12	8.22	5.4	18.6	33.44	2.32	171.2
13	9.35	5.8	18.6	27.59	2.37	193.6
14	8.79	5.4	19.8	27.96	2.52	182.5
15	8.22	6.6	16.2	34.43	2.00	171.7
16	7.65	6.2	17.4	35.83	2.13	161.4

**Table 4:** Analysis results on the effect of the design variables on the pressure drop.

	Level 1	Level 2	Level 3	Level 4	Deviation	%	Rank
*x*_1_	−44.21	−44.68	−45.22	−45.75	1.54	95.7	1
*x*_2_	−44.99	−44.96	−44.95	−44.97	0.03	1.8	3
*x*_3_	−44.99	−44.95	−44.97	−44.96	0.04	2.5	2

Total					1.61	100	

**Table 5:** Analysis results on the effect of the design variables on the maximum stress.

	Level 1	Level 2	Level 3	Level 4	Deviation	%	Rank
*x*_1_	−31.01	−30.25	−29.55	−29.07	1.93	61.7	1
*x*_2_	−29.96	−29.98	−29.93	−30.01	0.09	2.8	3
*x*_3_	−30.50	−30.06	−29.92	−29.39	1.11	35.5	2

Total					3.13	100	

**Table 6:** Analysis results on the effect of the design variables on the mass.

	Level 1	Level 2	Level 3	Level 4	Deviation	%	Rank
*x*_1_	−6.896	−6.997	−7.101	−7.203	0.307	13.7	2
*x*_2_	−7.067	−7.060	−7.046	−7.024	0.043	1.9	3
*x*_3_	−6.105	−6.736	−7.366	−7.991	1.886	84.4	1

Total					2.236	100	

**Table 7:** Comparison of the performance between the initial and optimal models.

	Stress (MPa)	Mass (kg)	Δ*p* (kPa)
Initial model	29.86	2.19	172.0
Optimal model	35.83	2.13	161.4
